# Supplementary material for: Metabolic dependency of chorismate in Plasmodium falciparum suggests an alternative source for the ubiquinone biosynthesis precursor
Source: Sci Rep. 2019 Sep 26;9:13936. doi: 10.1038/s41598-019-50319-5 (PMC6763611; doi:10.1038/s41598-019-50319-5)
Supplement: Supplementary file 1 — Supplementary Information [file 41598_2019_50319_MOESM1_ESM.pdf]

Supplementary Information

Metabolic dependency of chorismate in *Plasmodium falciparum* suggests an alternative source for the ubiquinone biosynthesis precursor

Ana Lisa Valenciano,[1] Maria L. Fernández-Murga,[2] Emilio F. Merino,[1] Nicole R. Holderman,[1] Grant J. Butschek,[1] Karl J. Shaffer,[3] Peter C. Tyler,[3] and Maria Belen Cassera[1*]

From the [1]Department of Biochemistry & Molecular Biology, and Center for Tropical and Emerging Global Diseases (CTEGD), University of Georgia, Athens, Georgia 30602, United States; [2]Laboratory of Experimental Pathology, Health Research Institute Hospital La Fe, Valencia 46026, Spain; [3]The Ferrier Research Institute, Victoria University of Wellington, Lower Hutt, New Zealand

* To whom correspondence should be addressed: Maria Belen Cassera: Department of Biochemistry & Molecular Biology, University of Georgia, Athens GA 30602; maria.cassera@uga.edu; Tel. (706) 542-5192.

Table S1. RPMI minimal and complete composition used in this study.

Nutrient	Final Concentration (μM or as indicated)	Minimal Medium (MM)	Complete RPMI Medium (CM)	Reagent Source
L-Arginine	200	+	+	Millipore-Sigma[1]
L-Asparagine	200	+	+	Millipore-Sigma
L-Aspartic acid	150	+	+	Millipore-Sigma
L-Cysteine	200	+	+	Millipore-Sigma
L-Glutamic acid	136	+	+	Millipore-Sigma
L-Glutamine	200	+	+	Millipore-Sigma
Glycine	133	+	+	Millipore-Sigma
L-Histidine	97	+	+	Millipore-Sigma
Hydroxy-L-proline	153	+	+	Thermo Fisher Scientific[2]
L-Isoleucine	100	+	+	Thermo Fisher Scientific
L-Leucine	381	+	+	Millipore-Sigma
L-Lysine	200	+	+	Millipore-Sigma
L-Methionine	101	+	+	Millipore-Sigma
L-Proline	174	+	+	Thermo Fisher Scientific
L-Serine	200	+	+	Millipore-Sigma
L-Threonine	168	+	+	Millipore-Sigma
L-Valine	171	+	+	Millipore-Sigma
Choline Chloride	0.0214	+	+	Millipore-Sigma
D-Biotin	0.00082	+	+	Millipore-Sigma
D-Calcium pantothenate	0.00052	+	+	Millipore-Sigma
Myo-Inositol	0.194	+	+	Millipore-Sigma
Niacinamide	0.0081	+	+	Millipore-Sigma
Pyridoxine hydrochloride	0.0048	+	+	Millipore-Sigma
Riboflavin	0.00052	+	+	Millipore-Sigma
Thiamine hydrochloride	0.00296	+	+	Millipore-Sigma
Vitamin B12	0.00037	+	+	Millipore-Sigma
Reduced Glutathione	3,254	+	+	Millipore-Sigma
$Ca(NO_3)_2$	609	+	+	Millipore-Sigma
HEPES	20,967	+	+	Thermo Fisher Scientific
KCl	5,365	+	+	Thermo Fisher Scientific
$MgSO_4$	407	+	+	Millipore-Sigma
NaCl	102,669	+	+	Thermo Fisher Scientific
Na_2HPO_4•7 H_2O	2,797	+	+	Thermo Fisher Scientific
D-Glucose	22,203	+	+	Millipore-Sigma
$NaHCO_3$	26,784	+	+	Millipore-Sigma
Gentamicin	41,876	+	+	Thermo Fisher Scientific
Hypoxanthine	367	+	+	Millipore-Sigma
Albumax II	5 (g/L)	+	+	Thermo Fisher Scientific
**Folic Acid**	**2.2**		+	Millipore-Sigma
***p*-Aminobenzoate**	**7.3**		+	Millipore-Sigma
**L-Phenylalanine**	**90**		+	Millipore-Sigma
**L-Tryptophan**	**24.5**		+	Millipore-Sigma
**L-Tyrosine**	**111**		+	Millipore-Sigma

[1] Burlington, MA, USA
[2] Waltham, MA, USA

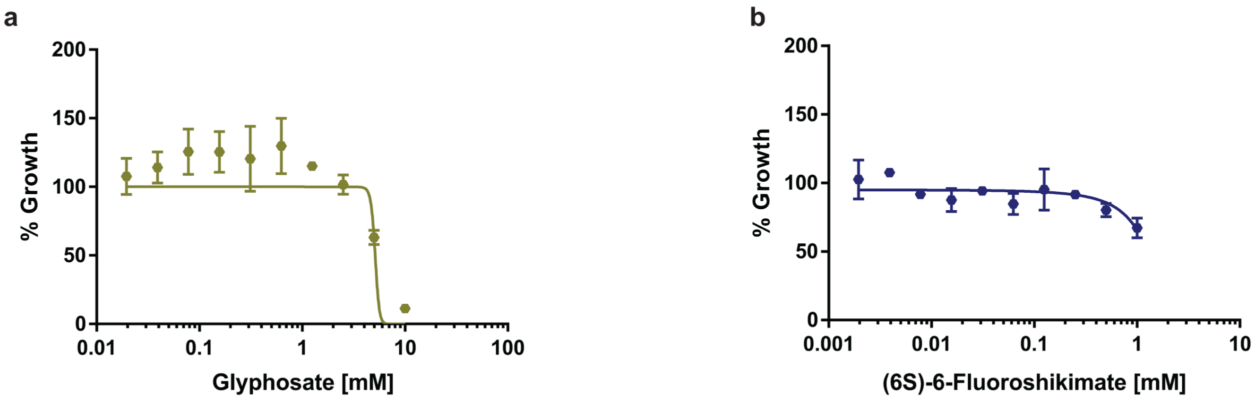

Fig. S1. Effect of glyphosate (panel a) and (6*S*)-6-fluoroshikimate (panel b) on *in vitro* growth of *P. falciparum*. Dose-dependent growth inhibition was determined after incubation for 72 h in the presence of increasing concentrations of the inhibitor in MM. Parasite growth was assessed by SYBR green. Results represent means ± s.e.m. of two independent assays.

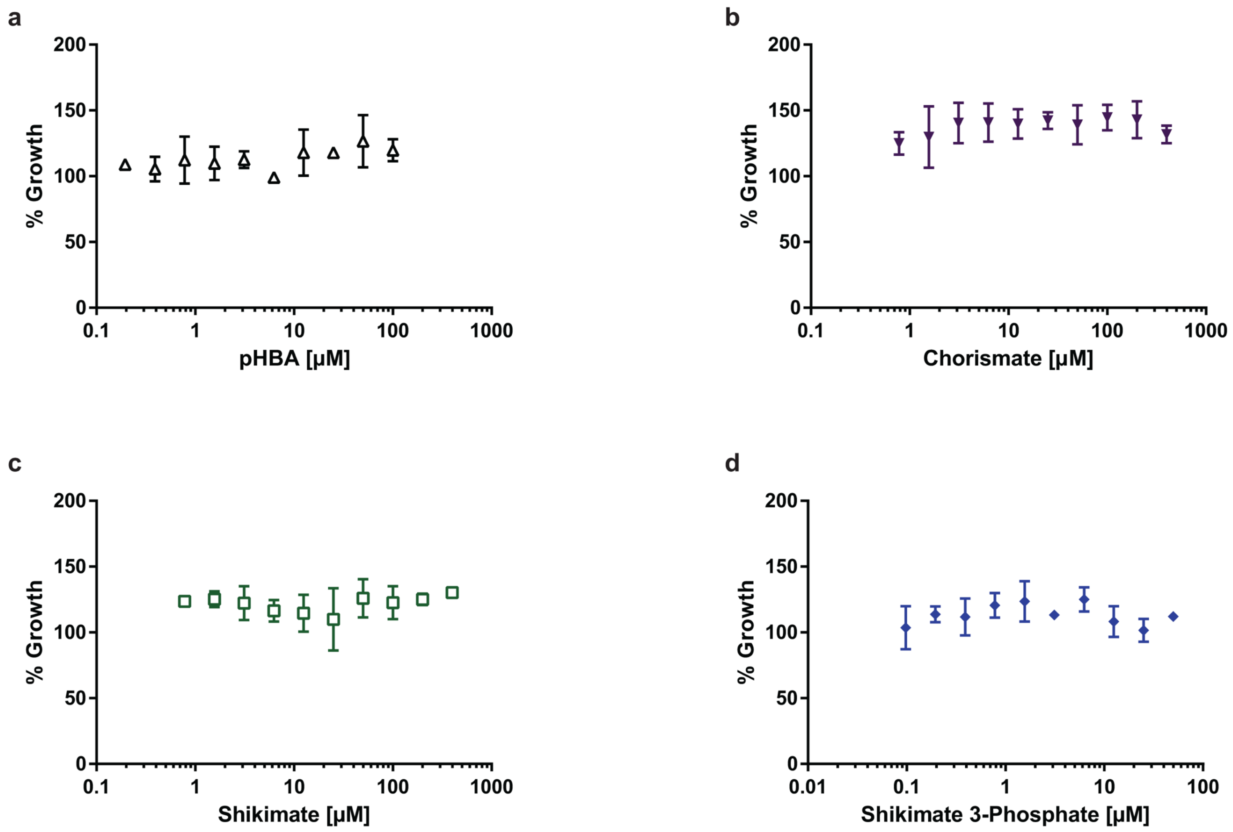

Fig. S2. Effect of metabolites on *P. falciparum in vitro* growth. Concentration-dependent metabolite toxicity or growth promotion was assessed in MM by SYBR green assay after 72 h incubation. Values represent means ± s.e.m. of at least three independent assays.

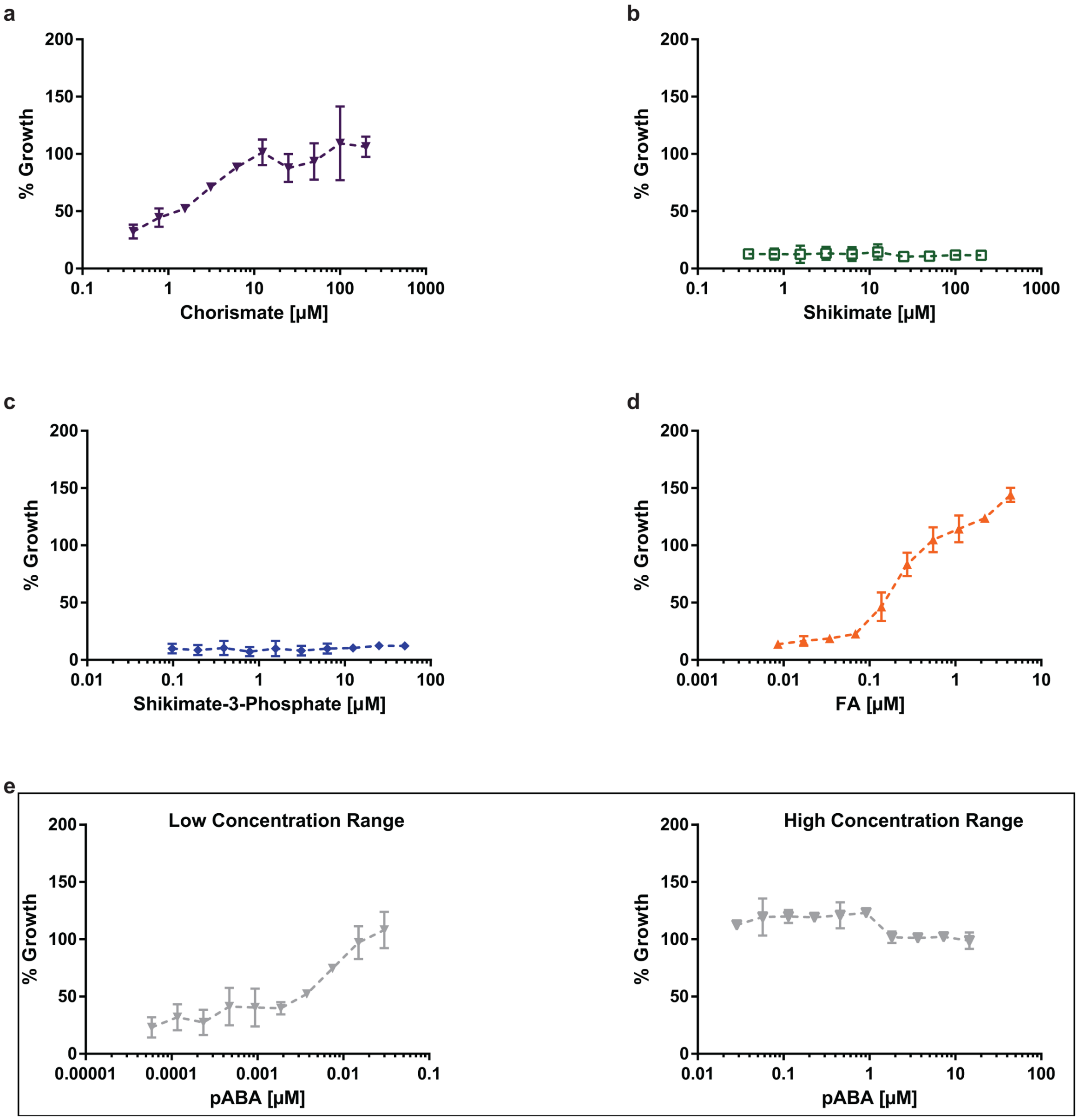

Fig. S3. Concentration dependence of metabolite rescue. *P. falciparum in vitro* reversal of growth inhibition by the indicated metabolites was performed in the presence of 0.25 µM MMV688345 in MM and increasing concentrations of metabolites as follow: **a)** chorismate (0.39-200 µM), **b)** shikimate (0.39-200 µM), **c)** shikimate-3-phosphate (0.098-50 µM), **d)** FA (0.0086-4.4 µM), **e)** pABA-low range (0.000059-0.03 µM) and pABA-high range (0.029-14.6 µM). Growth was assessed by SYBR green assay after 72 h incubation. Values represent means ± s.e.m. of at least three independent assays.

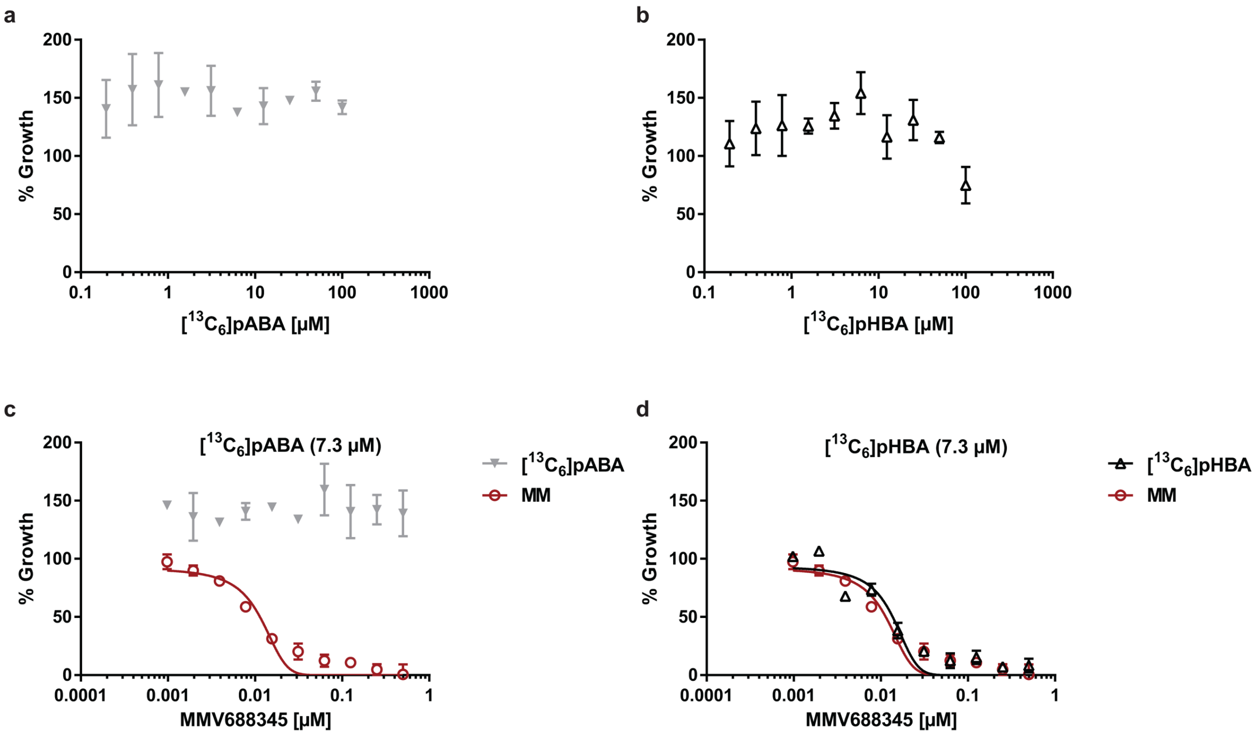

Fig. S4. Metabolite toxicity and reversal of MMV688345 growth inhibition by [$^{13}C_6$]pABA and [$^{13}C_6$]pHBA. Panels a and b: Concentration-dependent metabolite toxicity or growth promotion was assessed in MM by SYBR green assay after 72 h incubation. **Panels c and d:** *P. falciparum in vitro* reversal of growth inhibition by MMV688345 in the presence of 7.3 µM [$^{13}C_6$]pABA or [$^{13}C_6$]pHBA was assessed in MM by SYBR green assay after 72 h incubation. Values in all panels represent means ± s.e.m. of at least three independent assays.

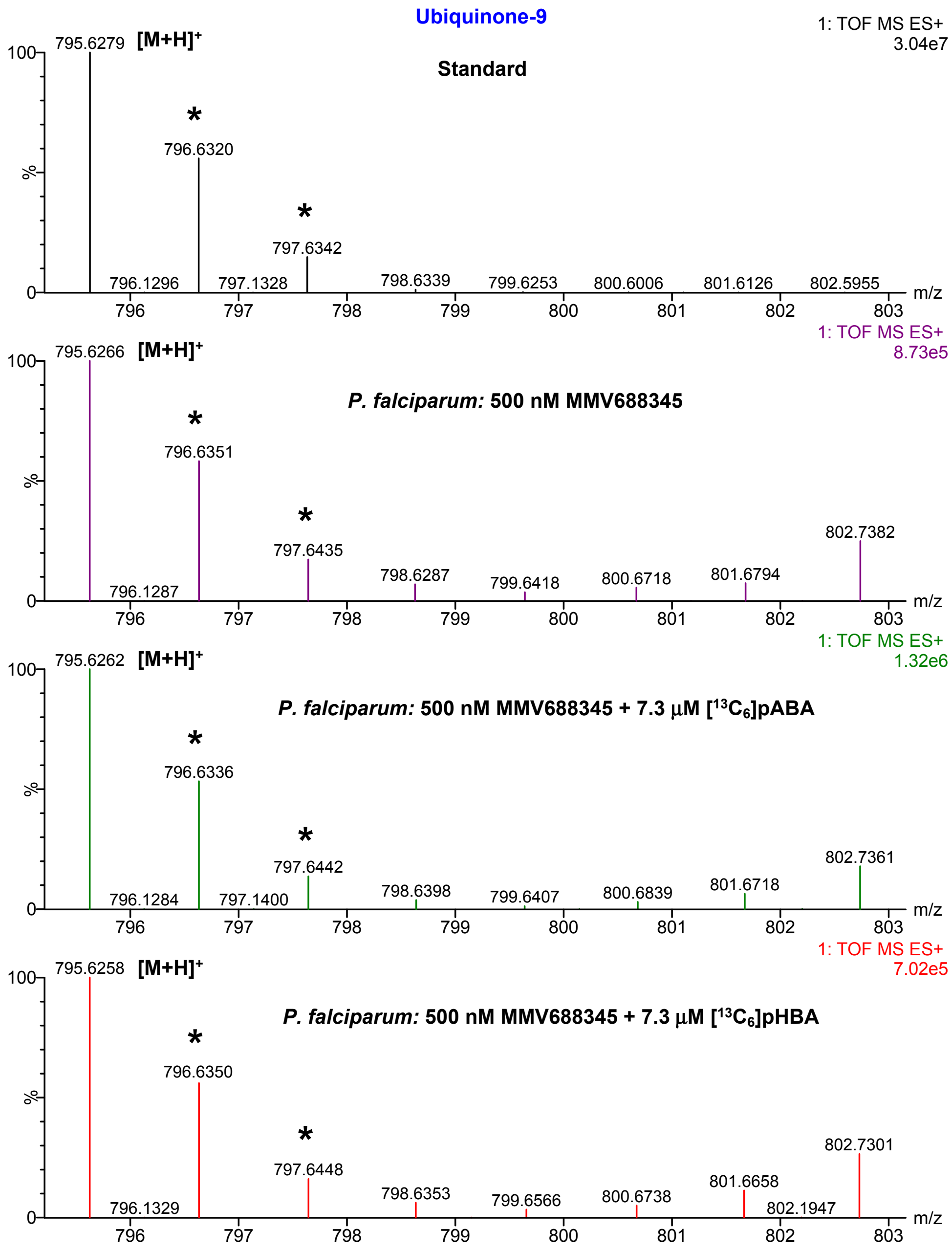

Fig. S5. LC-HRMS positive-ion mode spectra of ubiquinone-9 to assess whether *P. falciparum* is able to use [$^{13}C_6$]pHBA and [$^{13}C_6$]pABA as a metabolic precursor for ubiquinone biosynthesis. $[M+H]^+$ indicates the positive-ion corresponding to the mass of ubiquinone-9 and (*) indicates its natural isotopic distribution. The ion corresponding to the [$^{13}C_6$]ubiquinone-9 ($[M+H]^+$ expected = 801.6487) for [$^{13}C_6$]pHBA or [$^{13}C_6$]pABA incorporation into the head group of ubiquinone-9 was not detected.

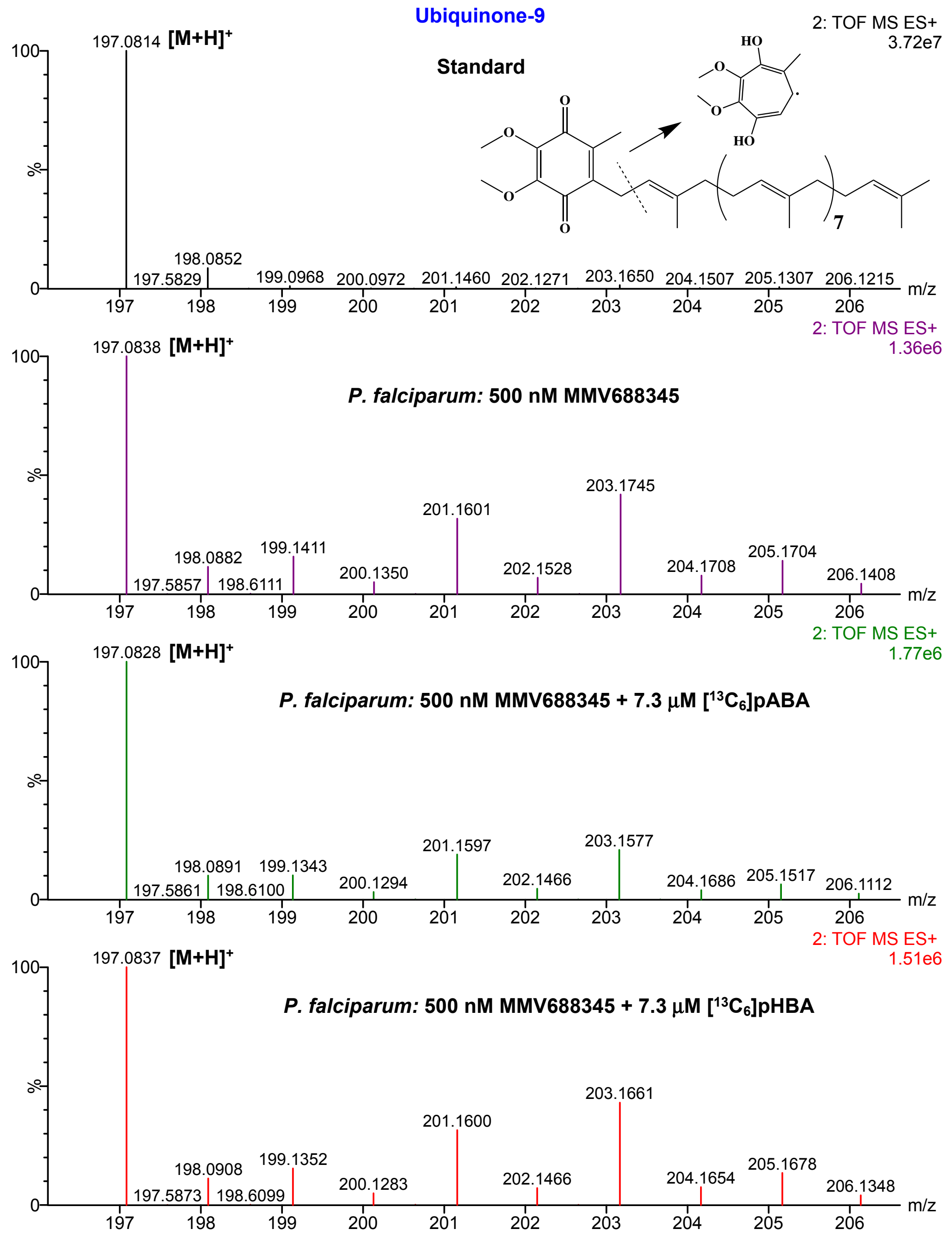

Fig. S5. (continuation) Mass fragmentation profile of ubiquinone-9. $[M+H]^+$ indicates the positive-ion corresponding to the mass of the ubiquinone-9 tropylium ion ($[M]^+$ expected = 197.0808). The predicted fragmentation is shown. The $[^{13}C_6]$tropylium ion ($^{13}C_6$-$[M]^+$ expected = 203.1010) was not detected.

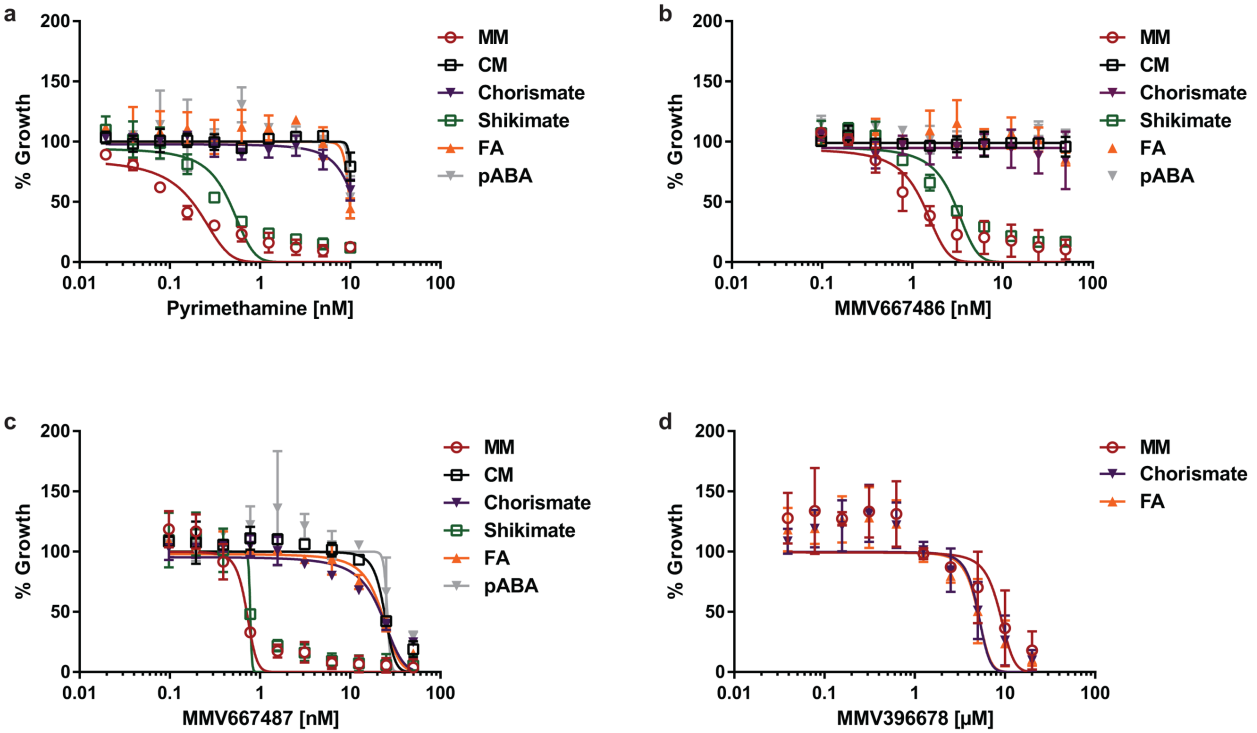

Fig. S6. Dose-dependent growth inhibition and growth recovery by metabolite supplementation. Growth inhibition observed in MM by pyrimethamine (IC_{50} = 0.18 ± 0.03 nM) **(panel a)**, MMV667486 (IC_{50} = 1.28 ± 0.17 nM) **(panel b)** and MMV667487 (IC_{50} = 0.71 ± 0.05 nM) **(panel c)** was reversed in CM while growth inhibition by MMV396678 (IC_{50} = 8,723 ± 1.3 nM) was not reversed by FA **(panel d).** Chorismate, FA and pABA but not shikimate, also reversed growth inhibition by pyrimethamine, MMV667486 and MMV667487 similar to MMV688345. The following metabolite concentrations were used: 25 µM shikimate, 12.5 µM chorismate, 2.2 µM FA and 7.3 µM pABA. Values represent means ± s.e.m. of at least three independent assays.
